# Biologics in Paediatric Crohn's Disease

**DOI:** 10.1155/2011/287574

**Published:** 2011-11-17

**Authors:** Oliver Gouldthorpe, Anthony G. Catto-Smith, George Alex

**Affiliations:** ^1^Department of Paediatrics, Monash University, Clayton, VIC 3800, Australia; ^2^Department of Gastroenterology and Clinical Nutrition, Royal Children's Hospital, Parkville Melbourne, VIC 3052, Australia; ^3^Gut and Liver Group, Murdoch Childrens Research Institute, Parkville, VIC 3052, Australia; ^4^Department of Paediatrics, University of Melbourne, Melbourne, VIC 3052, Australia

## Abstract

Crohn's disease affects increasing numbers of children worldwide. Generally, childhood-onset disease runs a more severe course than in adults and has a greater impact on quality of life. Therapy in children must take account of a different set of risks for toxicity compared to adults, but also to their longevity. Biologic drugs present remarkable advantages in terms of disease control for children, especially in those whose disease cannot be controlled with conventional therapies, but their long-term risks are still being assessed. Data regarding biologic use in children is limited and mostly amounts to case series, but results have been promising, both in terms of controlling disease activity and improving growth parameters. Adverse reactions are infrequent in the short term, but loss of response is a long-term problem, particularly in children. More information is needed about very long term risks. Infliximab and adalimumab are the most studied agents in children, while there is relatively limited data on certolizumab and natalizumab. Further collection of data on these agents is still needed, but this should not restrict access to these agents for children in whom no other agent is effective.

## 1. Introduction

The last 20 years have seen an evolution in the approach to therapy of Crohn's disease in both children and adults. The further development of ASA-based drugs, introduction of immunomodulators, and adoption of a “top-down” approach to early disease have brought substantial therapeutic benefit. However, persisting treatment failures lead to the development of a new class of drugs—biologicals—which have been used to target specific cytokines and receptors thought to be pivotal in the perpetuation of intestinal inflammation. Although a number have been developed, only a few have proven efficacy and entered standard clinical practice. A substantial proportion of patients develop Crohn's disease in childhood, but there are few studies of efficacy or safety of these new biologicals in this vulnerable population. The purpose of this paper is to summarise the information available on the efficacy and risks of available biologicals in childhood Crohn's disease.

## 2. Background

The incidence of childhood Crohn's disease appears to be increasing globally [1, 2]. The cause of this increase remains elusive, but it is now recognised to be occurring in both developed and developing nations [1] and in adults [3]. Crohn's disease, particularly in youth, results in significant morbidity, impairment in quality of life [4], and consumption of health care resources [5]. Children and their families especially feel its impact on their productivity [6]. Gender ratios differ from adults, with a male to female ratio of 1.5 to 1.0 in children less than 15 years of age compared to an equal gender ratio in adults with Crohn's [7].

Childhood-onset Crohn's disease tends to be characterized by ileocolonic or colonic involvement at outset [8, 9] rather than isolated ileal disease, as is seen more often in adults. Isolated colonic involvement is particularly apparent in early-onset paediatric disease [9, 10], as is isolated oral or perianal disease [9]. Diffuse involvement of multiple segments is common in children compared to the 1-2 localized segments more frequently seen in adults [8, 9, 11–13]. Upper gastrointestinal involvement is also more frequent in children at diagnosis than in adults. Disease extension tends to be more common in the first decade after diagnosis in children, although time to first surgery is delayed compared to adults [9, 13]. Children generally present with purely inflammatory disease, as opposed to the often penetrating or stricturing presentations seen in adults [8, 9]. Disease progression is also altered, with children developing complicated disease at a greater rate, despite increasing use of immunomodulators [8]. After 5 years of disease, the incidence of complicated disease is similar for children and adults [9]. This is an especially devastating observation, which is compounded by the simple fact that children spend more of their life at risk of these complications.

The genetics of paediatric CD has specific differences, mirroring its phenotypic distinctions. While there are many susceptibility loci common to adults and children, five new loci associated with childhood-onset disease have recently been identified through genome-wide association studies [14]. A full understanding of genes at these loci may be years away, and it remains unclear what impact these discoveries will have on clinical practice (if any) [15].

A growing pharmacopoeia of biologic drugs has broadened the treatment arsenal in the last fifteen years. In children, the two phases of treatment are induction and maintenance of remission. In the past, medical treatment options were limited. Induction mainstays have been corticosteroids and enteral nutrition [16]. Maintenance therapies include thiopurines, methotrexate, antibiotics, aminosalicylates, and enteral therapy [17–19]. There is some evidence that, while the acute responses to steroids is similar for adults (80–84%) and children (84–89%), a prolonged response may be more likely to be achieved in children (50–61% versus 32–44%) [20]. This may be related to a greater proportion of children who receive early immunomodulators. Despite this, there is still a significant proportion of children who fail these therapies altogether, experience significant toxicity, or become dependent upon corticosteroids [21]. In these children with otherwise refractory disease, biological agents have opened new horizons and become a mainstay of therapy, with important implications for improved growth and bone health. Indeed, there is evidence that the utility of a biological may be greater in children than in adults. At least one clinic which treats both adults and adolescents with inflammatory bowel disease has reported using biologicals in a substantially greater proportion of adolescents (20%) than adults (8%) [22]. 

Biologic drugs leverage components of the human immune system to target molecules implicated in the pathogenesis of Crohn's [23]. Most target the proinflammatory cytokine tumour necrosis factor alpha (TNF*α*). Infliximab (Remicade) was the first drug of this class, synthesised as a mouse-human chimera [24]. A fully humanised molecule, adalimumab (Humira), and certolizumab (Cimzia), a polyethylene-glycolated antibody-binding fragment, have since followed. Natalizumab (Tysabri), an *α*-integrin monoclonal antibody, has also been used in the management of paediatric CD.

Most trial evidence for the use of biologics comes from adults, and while there is more similarity than difference between adults and children, this literature must be interpreted with caution [25, 26]. Given the more severe phenotype, duration, and genetic differences of paediatric-onset CD, it may be that an entirely different therapeutic approach is warranted. Efficacy studies examining the early use of immunomodulators such as thiopurines in children [27] have formed the basis of what is now known as “top-down” therapy [28]. This term was coined to define “aggressive initial therapy” with “disease modifying agents” [28], and is currently considered to encompass early use of either or both biologicals and immunomodulators [20]. There is some evidence which points to the possible efficacy of “top-down” therapy with biologicals, where children identified as likely to have progressive disease are aggressively treated with early immunomodulators and/or biologics [29]. The difficulty lies in identifying those whose disease is likely to run a more aggressive course, for whom potential benefits should outweigh risks.

Disease activity indices used in children are different from those used in adults, adding a further layer of complexity. Since Crohn's disease can have a substantial impact on growth, the Paediatric Crohn's Disease Activity Index (PCDAI) includes height and weight criteria [30]. It is weighted more towards objective laboratory values when compared with the adult Crohn's Disease Activity Index (CDAI) [31]. The PCDAI has been shown to be a responsive, well-validated index with good reliability [32]. It can be used to establish baseline severity and to define treatment outcomes such as partial treatment response or complete remission. Definitions of secondary loss of response to treatment vary, but this is generally considered to have occurred if a child does not remain in remission, as defined by the PCDAI. The importance of mucosal healing in the long-term prognosis of disease is well acknowledged and should also be considered as an outcome measure [33].

## 3. Clinical Efficacy

There is a paucity of authoritative evidence regarding the use of biologics in paediatric CD. Infliximab is the main agent used, with other biologics used mostly in the event of lost response to infliximab therapy. There are no trials comparing the relative efficacy of one biologic with another. Paediatric evidence regarding the use of biologics in the management of fistulising and postsurgical patients is scant.

### 3.1. Infliximab

Randomised controlled trial evidence regarding the use of infliximab is confined to adults. Here, infliximab has proven efficacy for induction, maintenance, and for the treatment of fistulae [34–36]. For induction in children, efficacy is inferred from Level 3 and 4 evidence [37]. In these studies, the rate of successful induction is approximately 88%. In early paediatric pilot studies, induction of remission was seen first with a single dose, then in a multiple-dose induction regimen of doses at zero, two, and six weeks [38, 39]. The larger REACH study in children [40] showed higher rates of successful induction than in many adults. For example, in a somewhat comparable group receiving both azathioprine and infliximab, the rate of steroid-free remission was only 47% after induction [41].

For maintenance, the REACH study also provides the best support for the use of infliximab in children [40]. In this multicentre, open-label study, 112 children (median age: 13 years) were all given three doses of infliximab for induction. At ten weeks, all were assessed for treatment response. Those who responded were then randomised to receive further maintenance treatment with infliximab, either every eight weeks or every twelve weeks. Participants were followed for a total of fifty-four weeks. Response and remission were defined with appropriate PCDAI values.

At ten weeks, 88.4% had responded and 58.9% entered remission. At fifty-four weeks, in those receiving infliximab every eight or twelve weeks, respectively, 55.8% and 23.5% remained in remission. These data suggest that infliximab does have some efficacy in children. However, the REACH study has significant limitations, including the lack of a control group during induction, the universal use of concurrent immunomodulators, the unblinded nature of the study, randomization of participants to a dose frequency which was almost certain to be inferior, and, extensive involvement of the manufacturer in the design, data acquisition, and authorship of the study. These limitations obfuscate the true size of the treatment effect. They also add further difficulty when making comparisons with adult data. The only therapeutic property of infliximab that this study proves beyond contention is that it should be given every eight weeks for maintenance in children.

The possibility of using infliximab only in episodes of exacerbated disease was raised due to concerns about minimising exposure to biologics in children. The study by Ruemmele and colleagues confirms that scheduled doses are superior to episodic therapy in children, at least in terms of treatment effect [42]. Forty children were given open-label infliximab induction. Those who entered remission (*n* = 34) were then randomised to regular infliximab every eight weeks or to receive infliximab only should they develop an exacerbation. After sixty weeks of total followup, those who had received scheduled maintenance were more likely to be in remission (83% versus 63%; *P* = 0.011); however, the study's methodology limits the significance of these results. Of most concern, the study used the Harvey-Bradshaw Index (HBI), which is based on, but correlates poorly with, the CDAI [43, 44]. Neither the CDAI nor the HBI has been validated for use in children. In any case, most clinicians now use infliximab on a scheduled basis in maintenance.

Secondary loss of response to infliximab is a major limiting factor in maintenance therapy. It may require dose escalation or the cessation of infliximab therapy. Within the first year, in adults with luminal disease, approximately 30% will lose response [35]. In children with luminal disease, a similar number also lose response (36.5% in the REACH study) [40]. Beyond the length of followup possible with RCTs, cohort studies provide important information about the long-term durability of infliximab therapy. There appears to be no upper limit to the proportion of patients who will experience secondary loss of response. In one cohort of children receiving maintenance infliximab, only 33% were still in remission (not requiring corticosteroids or surgery) by three years [45].

There appears to be some correlation between the rates of secondary loss of response and the acquisition of antibodies to infliximab, in both adults and children [35, 46, 47]. The rate of secondary loss of response may be reduced with scheduled maintenance therapy and the use of concurrent immunomodulators such as methotrexate or thiopurines; however, these should be used with caution in children, due to concerns about the possible risks for malignancy (see the section “Adverse Effects,” below) [46, 48, 49]. 

The early and aggressive use of infliximab in selected paediatric patients is a nascent area of research. The top-down approach to therapy supposes that early disease (commonly possessing inflammatory behaviour) is the most amenable to early aggressive therapy such as with immunosuppressants or biologicals, given that infliximab, for instance, targets the inflammatory cascade [29]. Indeed, children with early disease display immunological features that disappear later in the course of disease [50]. By intervening early, it may be possible to prevent progression to stenosing or penetrating disease (so-called disease modification). 

In children, Kim and colleagues address the issue of top-down therapy with infliximab in a case series [51]. The study took eleven patients resistant to conventional therapy (step-up group) and eighteen treatment naïve patients with moderate to severe disease (top-down group). All were given infliximab induction and maintenance therapy for one year. More patients in the top down group were in remission after induction, and after ten months of maintenance therapy. The limitations of this study are clear, but the possible superior efficacy of top-down therapy in children cannot be excluded at this stage. 

The key issues in top down therapy, which remain unresolved, are to reliably indentify those patients who will go on to experience complications, so warranting the risks of aggressive therapy [29], as well as those who will receive therapeutic benefit from biologicals [52]. 

### 3.2. Adalimumab

Again, randomised controlled trial evidence for the efficacy of adalimumab is confined to adults. Here it has been proven effective in both the induction and maintenance of remission [53–55]. These studies include patients who are both naïve to biologics and those who have failed infliximab therapy. Adalimumab has also been shown to reduce hospital admissions and CD-related surgery [56]. As with other biologics, there appears to be a considerable rate of secondary loss of response, with one case series describing a 21% cumulative probability by one year [57]. We await the published results of a large, double-blind, paediatric study listed as complete on http://www.clinicaltrials.gov/ct2/show/NCT00409682?term=NCT00409682—this study and results are available in abstract form.

In children, limited prospective evidence comes from the study by Viola and colleagues [58]. This case series was conducted in relatively older patients (median age: 16 years) with moderate to severe CD. Twenty-three patients received adequate loading doses at zero and two weeks and then went on to receive forty-eight weeks of maintenance. 61% were in remission after induction, and 65% were in remission by the end of the study. Immunomodulators and corticosteroids were used as clinically indicated during the study, and requirements were significantly lower at the end of the study.

There is retrospective evidence that supports the use of adalimumab in children. The RESEAT cohort reports outcomes in 115 children (mean age at diagnosis: 11 years) who had almost all previously received infliximab [59]. Loss of response or adverse reaction to infliximab had occurred in 92% of subjects. By one year, the rate of clinical remission was 42%, demonstrating some efficacy of adalimumab as a rescue therapy for children who fail infliximab. A survey of UK and Ireland clinicians describes seventy-two patients and reports a higher rate of remission (61%), but differing baseline patient characteristics and study methodologies make it very difficult to compare these two studies [60]. A smaller Israeli cohort (*n* = 14) also demonstrated efficacy in children with severe treatment refractory CD [61].

### 3.3. Certolizumab

Certolizumab shows evidence of moderate efficacy in adults with CD [62–64]. There are currently no published studies in children regarding the efficacy of this drug, but two trials are currently in progress (NCT00899678, NCT01190410).

### 3.4. Natalizumab

Natalizumab was first developed for use in multiple sclerosis. The ENCORE trial demonstrated moderate efficacy in the induction of remission in adult Crohn's [65]. Hyams and colleagues conducted a small case-series with the primary aim of assessing the safety of natalizumab in adolescents with Crohn's [66]. In secondary analysis, eleven of thirty-eight patients were in remission at ten weeks. The paediatric use of this drug should be investigated further; one trial in children aged 12–17 years is listed on ClinicalTrials.gov as complete, but data is yet to be published (NCT00055367).

## 4. Growth and Bone Health

Families and children with CD are often concerned about the physical and psychosocial impact of impaired growth. While growth arrest may be a central feature early in the course of disease, it appears that most children will go on to achieve an adult height that is within normal limits [67–69]. Nonetheless, around one-fifth will have impaired adult height, with late diagnosis and jejunal disease being predictive factors [69]. When managing growth in CD, the three general aims are to control disease activity, optimise nutrition, and minimise the use of corticosteroids (but not at the expense of poor disease control) [26, 67, 70]. 

Growth failure mostly appears to be due to disease activity, with smaller nutritional and iatrogenic components [67]. Growth and pubertal development are inexorably linked around the time many children develop CD. A growing body of research describes the complex milieu of cytokines that modulate growth and development in Crohn's [71]. There appears to be a variety of growth impairment phenotypes; for example, children demonstrate various growth hormone and insulin-like growth factor-1 (IGF1) deficiency states [72]. This causes difficulty in identifying possible therapeutic targets. Translational research implicates interleukin-6 and TNF*α* in the suppression of the growth hormone axis, by inhibiting hepatocyte IGF1 production [73, 74]. In vitro interleukin-1 and TNF*α* both inhibit activity at the growth plate in long bones [75].

By targeting TNF*α* directly, the anti-TNF*α* class of medications may improve growth. The REACH study reported height data only for children with a bone age delay of at least one year and did not report on a Tanner stage [40]. *z*-scores (the number of standard deviations above or below the mean) were calculated from age- and sex-matched population values. At baseline, the mean *z*-score for height was −1.5. At fifty-four weeks, the mean improvement in *z*-score was 0.5 (*P* < 0.001). Despite this impressive result, given the likely high incidence of pubertal delay in this group, comparing them with healthy age-matched controls is of limited value [76]; that is, the improvement in height may be due to a treatment effect or simply reflect a relative difference in unmodified growth velocities between the treatment group and the population at large.

Three retrospective studies have since provided limited, yet more compelling, evidence of infliximab's efficacy in growth. The first describes twenty-seven children (18 male) with otherwise refractory Crohn's who received maintenance infliximab [77]. It found that height and height velocities are most improved when treatment is initiated before or during early puberty (Tanner I-III). Treatment has a lesser effect on growth later in puberty (Tanner IV, V). This emphasises that timing is critical in gaining control over disease, at least as far as height is concerned. The second study described twenty-eight children (17 male) who were retrospectively assessed over an eighteen-month period, during which time they all received induction therapy. Some also received maintenance therapy (*n* = 12) [78]. In patients who responded to infliximab (75%), mean improvement in height velocity was 4.4 cm/year, compared to non-responders whose height velocity remained static. While many progressed into puberty during the study, children who remained prepubertal also had improvements in growth. This suggests that growth is improved independent of any permissive effect infliximab has on progression into, or through, puberty. In both studies, there were a proportion of patients who had sustained growth impairment. This further emphasises the heterogeneity of growth impairment phenotypes. Finally, there is some evidence that increasing infliximab dosing frequency may further improve growth [79].

Quite apart from the effect of Crohn's disease on growth, bone health is impaired, with bone deficits being well documented in children [80, 81]. Therapy with infliximab results in dramatic improvements in biomarkers of bone formation [82], consistent with the reversal of a direct effect of TNF*α* on the balance of bone formation and resorption. TNF*α* is known to inhibit osteoblast differentiation, inhibit osteoblast collagen secretion, and induce osteoblast apoptosis [83, 84].

The use of biologics in growth impairment warrants further investigation. There are no published data regarding the growth properties of the other anti-TNF*α* agents or natalizumab. Given that growth is unique to paediatrics, here more than in any other areas, randomised controlled trials in this area are needed.

## 5. Adverse Effects

### 5.1. Anti-TNF*α* Agents

There are a number of toxicities that may occur in anti-TNF*α* therapy. These include infusion reactions, opportunistic infections, dermatological conditions, and malignancy. Most data comes from infliximab use, and, until more paediatric data are available, these should be extrapolated to other drugs of the anti-TNF*α* class. Thus, close monitoring is required when using any anti-TNF*α* agent in children.

Infusion reactions occur commonly with the use of infliximab and may be severe in some cases. In REACH, 17-18% of children had infusion reactions, while only one infusion reaction was severe enough to warrant cessation of infliximab [40]. Retrospective review of children receiving infliximab infusions at a single centre showed that female gender and short duration of immunomodulator use were risk factors for a reaction [85]. It is possible that children experiencing genuine anaphylactic reactions may be able to be desensitized through graduated reexposure to an anti-TNF*α* agent [86, 87]. The rate of further severe reactions is considerable [85, 87]. Reintroduction should be considered a second-line approach now that alternative biologics are available. Although patients are often predosed with antihistamines and steroids before infusions, there is relatively little evidence, at least for antihistamines, that these provide significant protection against acute infusion reactions in children [88].

A large number of children develop antinuclear antibodies (ANAs) after infliximab exposure [40], and to date there has been at least one reported case of drug-induced lupus in a child [89]. Data regarding ANA seroconversion in children exposed to other anti-TNF*α* agents CH is unavailable. The development of other autoimmune diseases, such as demyelinating disease, has not been observed to date in children with Crohn's disease although it has occurred in children given infliximab for other indications.

Serious and/or opportunistic infections are unusual, but do occur. Adult studies demonstrate similar rates between placebo and treatment groups, and this is compatible with the available uncontrolled paediatric data [90]. In REACH, where most patients were receiving an immunomodulator and some were receiving corticosteroids, 8% of patients experienced a serious infection [40]. Two deaths from sepsis have been reported in children receiving adalimumab in the context of multiple immunosuppressants and central venous lines [60]. Concurrent use of anti-TNF*α* agents and immunomodulators, particularly in combination with corticosteroids, is a recognised risk factor for infection [91]. Cohort studies of children receiving adalimumab and infliximab have both reported low rates of infection [45, 59]. Reactivation of latent *Mycobacterium spp.* infections is of major concern in children with endemic exposure. One such case is reported in a cohort of children receiving infliximab [45]. Immunomodulators affect the sensitivity of immunological tests for *M. tuberculosis,* so all children should be screened appropriately before starting therapy [92]. An added risk with anti-TNF therapy is the reactivation of chronic viral infection, particularly hepatitis B and herpes zoster [93].

As with adults [94], there is general agreement with the importance of screening before embarking on therapy with an anti-TNF in children. This should include a careful history for risk factors for tuberculosis [95]. Anergy can limit the value of skin testing with PPD [92], and in vitro specific blood tests may be more sensitive and specific [96], though there is little information in children. Active infection—either bacterial, mycobacterial, or viral—poses a particular risk for exacerbation or dissemination of sepsis in adults [97, 98] with obviously similar risks in children. Immunization histories are important to obtain, and it may be necessary to confirm that protective titres are present. If appropriate, some immunizations may need to be repeated. The severity of varicella zoster infections in patients on infliximab [97] argues strongly for including this in immunization. Given the increasingly early use of immunomodifiers, immunization status should be established and appropriately brought up to date at the first diagnosis of inflammatory bowel disease in a child. This anticipatory approach should also include annual influenza vaccination, pneumococcal vaccination, human papillomavirus vaccination to young females, and hepatitis B to seronegative patients [99]. A history of malignancy is also recognised to pose a risk factor [95]. There are significant disparities between countries in the practice and extent of screening before commencing a biological in children [95]. 

An additional risk for infants is the biological impact of placenta-fetal passage of anti-TNF therapies. A death after vaccination from disseminated *Bacillus* Calmette-Guerin's infection has been reported in a 3-month-old otherwise healthy infant whose mother was being treated with infliximab for Crohn's disease [100].

A paradoxical dermatological effect is infliximab-induced psoriasis (IIP). IIP appears to occur in children of both sexes at a rate of 8–10% and to be primarily of the non-pustular subtypes [55, 101]. They may also occur more on the facial areas. This contrasts with adults, where lesions are predominantly of the palmoplantar pustular sub-type [102]. While these lesions are generally mild, their cosmetic impact may be of great significance to children and adolescents.

Malignancy can be considered a rare occurrence with the use of biologics at this stage. Any discussion of malignancy must be prefaced with mention of the high rate of malignancy in poorly controlled CD; for example, intestinal malignancy occurs at a rate of approximately 0.8 cases/1000 person years [103]. For colonic cancer, this is around two to three times normal, and, for small bowel cancer, there is an eighteenfold rate increase.

There is some evidence that treatment with anti-TNF agents may increase the general risk for malignancy [104]. However, of the malignancies in 48 children reported by Diak [104], interpretation was confounded by the potential risk for malignancy of the underlying condition and concomitant immunosuppression. Concerns have focussed on lymphoma. At least one meta-analysis of controlled trials using anti-TNF*α* agents in Crohn's found no increase in cancer risk overall when compared to placebo [105]. However, a meta-analysis that also included data from lower-level studies detected an increased rate of lymphoma in patients receiving anti-TNF*α* therapy, when compared to a reference population (standardized incidence ratio: 3.23; 95% confidence interval: 1.5–6.9) [106], but the detection of any increased risk of malignancy is sobering. It should be noted that there is no general association between inflammatory bowel disease and lymphoma [107]. One case of lymphoma is reported in a cohort of children receiving infliximab [45].

Hepatosplenic T-cell lymphoma (HSTCL) is an extremely rare but mostly fatal malignancy occurring almost exclusively in young males receiving thiopurines [108]. Of thirty-six reported cases in the CD literature, seventeen were also receiving anti-TNF*α* therapy. HSTCL risk can probably be mostly attributed to thiopurine exposure: in one study of thiopurine users, the at-risk age bracket was shown to be at a fourfold increased risk of lymphoma in general [109]. However, it is possible that the risk of HSTCL is additionally increased with the use of infliximab, and cases of HSTCL in CD patients receiving anti-TNF*α* monotherapy may yet emerge [108]. In the absence of data regarding treatment effect size of biologic therapy in children, it is extremely difficult to make evidence-based decisions about the risk of malignancy in poorly controlled disease compared with well-controlled disease in patients receiving thiopurines and biologic drugs. There is a good case to be made for using thiopurines as an adjunct to reduce the risk of loss of response to anti-TNF*α* agent in the first year of therapy. However, the long-term use of both immunomodulator and anti-TNF*α* agent should include careful and informed discussion between the family and physician, particularly taking account of the implications if the child should lose response to anti-TNF*α* therapy. The use of methotrexate as an alternative agent should be considered, although the risks of this as an adjunct are not well known. 

### 5.2. Natalizumab

There is only one study regarding the safety of natalizumab in children [66]. In this study, the short-term safety profile of the drug was assessed as broadly similar to that seen in adults. Long-term safety data for natalizumab in children is currently unavailable.

Progressive multifocal leukoencephalopathy (PML) is associated with polyomavirus JC reactivation in patients receiving natalizumab [110]. The risk is approximately 1 case/1000 patients [111]. It is mostly fatal, and reliable therapies are not available. After a temporary and voluntary withdrawal from the market, natalizumab therapy has been remarketed with strict limitations [112]. Of note in the context of CD management, there is a requirement for the cessation of all other immunomodulators. This likely renders the use of natalizumab impractical for most patients with CD. Extreme caution should be exercised when using this unproven therapy in children because of the risk of PML.

## 6. Access

Regulatory and funding arrangements constrain the use of biologicals in children. Here we consider the United States (US), European Union (EU), and Australian jurisdictions. Infliximab is the only drug with an explicit licence for use in children around the world. Adalimumab is approved for use in adults but not approved for use in children. In the United States, somewhat more permissive licensing has allowed for the use of certolizumab and natalizumab in adult CD. Approval has either been refused or not sought in the EU and Australia. Compassionate funding arrangements have permitted the off-label use of drugs other than infliximab in children.

## 7. Conclusion

There is a need for higher-quality evidence to inform decision making when using biologics in paediatric Crohn's. While it may be argued that children and their families will not accept the risks involved in such research, on the contrary there is no reason to accept that treatment is currently guided by little or no rigorous evidence specific to children [113]. It is not our intention to present a pessimistic assessment of biologicals—they have an important and valuable place in the management of paediatric Crohn's and their pragmatic use should continue. Future advancements and new drugs will come. These may open avenues of therapy for certain patients, but further gains may be made simply by deepening our understanding of current therapies.
